# Time of Flight in Perspective: Instrumental and Computational Aspects of Time Resolution in Positron Emission Tomography

**DOI:** 10.1109/trpms.2021.3084539

**Published:** 2021-05-27

**Authors:** Dennis R. Schaart, Georg Schramm, Johan Nuyts, Suleman Surti

**Affiliations:** Section Medical Physics & Technology, Radiation Science and Technology Department, Delft University of Technology, 2629 JB Delft, The Netherlands; Department of Imaging and Pathology, Division of Nuclear Medicine, KU/UZ Leuven, 3000 Leuven, Belgium; Department of Imaging and Pathology, Division of Nuclear Medicine, KU/UZ Leuven, 3000 Leuven, Belgium; Department of Radiology, University of Pennsylvania, Philadelphia, PA 19104 USA

**Keywords:** Image quality, image reconstruction, photo-detectors, scintillators, time-of-flight positron emission tomography (TOF-PET)

## Abstract

The first time-of-flight positron emission tomography (TOF-PET) scanners were developed as early as in the 1980s. However, the poor light output and low detection efficiency of TOF-capable detectors available at the time limited any gain in image quality achieved with these TOF-PET scanners over the traditional non-TOF PET scanners. The discovery of LSO and other Lu-based scintillators revived interest in TOF-PET and led to the development of a second generation of scanners with high sensitivity and spatial resolution in the mid-2000s. The introduction of the silicon photomultiplier (SiPM) has recently yielded a third generation of TOF-PET systems with unprecedented imaging performance. Parallel to these instrumentation developments, much progress has been made in the development of image reconstruction algorithms that better utilize the additional information provided by TOF. Overall, the benefits range from a reduction in image variance (SNR increase), through allowing joint estimation of activity and attenuation, to better reconstructing data from limited angle systems. In this work, we review these developments, focusing on three broad areas: 1) timing theory and factors affecting the time resolution of a TOF-PET system; 2) utilization of TOF information for improved image reconstruction; and 3) quantification of the benefits of TOF compared to non-TOF PET. Finally, we offer a brief outlook on the TOF-PET developments anticipated in the short and longer term. Throughout this work, we aim to maintain a clinically driven perspective, treating TOF as one of multiple (and sometimes competitive) factors that can aid in the optimization of PET imaging performance.

## I. Introduction

EVER since the reintroduction of time-of-flight positron emission tomography (TOF-PET) in the mid-2000s there has been a surge in activity related to hardware and computational developments that not only aim to further improve device performance but also utilize the precise timing information for improvements in image quality and clinical practice. This article provides a general review of TOF-PET, aiming to provide our perspective on the past, present, and future of the field. As such, it does not aim to cover the full spectrum of work in this area, for which several other review articles have been published.

### A. Rationale and Principle of TOF in PET

A valid signal in PET is determined by the coincident detection of a pair of almost back-to-back 511-keV photons that are produced in an electron-position annihilation event. The positron is emitted by a radiolabeled tracer previously administered to the patient. Detection of a pair of coincident photons provides an electronic collimation that defines the annihilation photon emission point to lie somewhere along the line connecting the two PET detectors. This line is called the line of response (LOR).

**Fig. 1.**
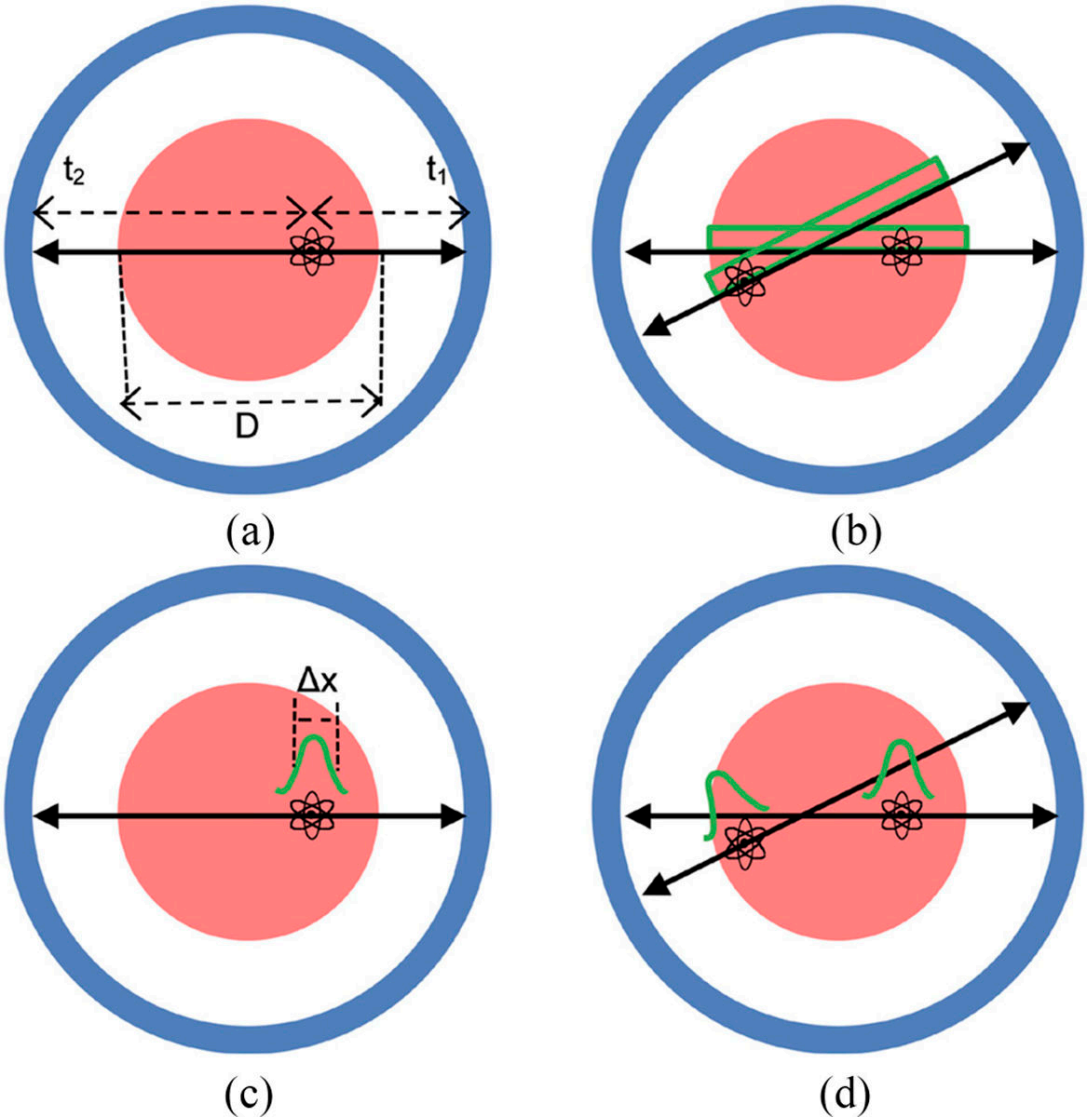
(a) Annihilation point occurring within an object of diameter D with the annihilation photons detected at times *t*_1_ and *t*_2_ in the PET scanner. (b) In a non-TOF scanner, a uniform location probability along the LOR is assumed for the emission point, leading to noise correlations in image reconstruction due to overlapping LOR bins. (c) With improved TOF, the emission point is better localized along the LOR, with a precision that is defined by a Gaussian distribution of width Δ*x*. (d) Improved localization of the two emission points along the individual LORs reduces the noise correlation in image reconstruction since emissions from two different voxels have reduced (or no, as shown here) overlapping TOF LOR bins.

The arrival times of the two annihilation photons have to lie within a predetermined coincidence time window, 2τ, which is normally set to cover the full imaging field of view (FOV). Typically, this will be set at ±2.5 ns to cover a 60 cm FOV in modern systems. The location of the emission point along the LOR is given by the difference in detection times, or TOF difference, of the two annihilation photons *t*_2_ − *t*_1_ [Fig. 1(a)]. In conventional, non-TOF PET the precision of TOF measurement (TOF resolution, or Δ*t*) is low (>1 ns) such that the emission point has a uniform probability to lie anywhere along the LOR within the object. However, collection of all LORs over the full azimuthal space is sufficient to provide an accurate tomographic image of all the emission points (or radiotracer distribution) using an image reconstruction algorithm [1]. The assumption of a uniform probability for location of an emission point along the full LOR length will lead to noise correlations since emissions from two different voxels will have overlapping LOR bins [Fig. 1(b)], thereby affecting the image signal-to-noise ratio (SNR) [2], [3]. Sub-ns TOF resolution in TOF-PET allows a more precise localization of the emission point along the LOR [Fig. 1(c)]. For a TOF resolution of 300 ps FWHM, this translates into a spatial uncertainty Δ*x* = *c*Δ*t*/2, with *c* the speed of light, of the emission point along the LOR of 4.5 cm FWHM. Noise correlations during image reconstruction are therefore limited to fewer voxels, as defined by the TOF resolution [Fig. 1(d)], and hence lead to variance reduction and thus improved image SNR [4]–[6].

### B. History of TOF-PET

The first generation of TOF-PET scanners were developed as early as in the 1980s when the primary application of PET was in brain and cardiac imaging using fast decaying isotopes [7]–[12]. These systems were based on cesium fluoride (CsF) or barium fluoride (BaF_2_) scintillators and achieved TOF resolutions in the range of 450–750 ps FWHM. However, the low detection efficiency and low light output of these crystals led to trade-offs in the system performance. The low detection efficiency of the crystal directly translated into a low intrinsic system sensitivity. The low light output of the scintillator required (near) one-to-one coupling of the crystal to a photodetector (photomultiplier tube, or PMT) in order to maintain good timing performance. However, the size of the smallest PMT (>1 cm) limited the system spatial resolution. Hence, the first generation TOF-PET systems were eventually eclipsed by the superior overall performance of bismuth germanate (BGO)-based scanners, despite their lack of TOF capability [13]. The discovery of cerium-doped lutetium oxyorthosilicate (LSO:Ce) [14] and other lutetium (Lu)-based scintillators in the mid to late 1990s first led to the replacement of BGO with these Lu-based crystals that had similar detection efficiency but higher light output (improved spatial resolution and fully 3D scanner design for improved sensitivity) and fast signal characteristics (reduced dead time) [15]. In parallel, it was recognized that these crystals could also be used in the development of TOF-PET systems [16], [17] without the limiting design trade-offs present in the first generation TOF-PET systems. This led to the development of a second generation of TOF-PET scanners in the mid-2000s with much higher system sensitivity and improved spatial resolution, while achieving TOF resolution in the 450–600 ps range [18]–[21]. More recently, the development of silicon photomultipliers (SiPMs) has led to the widespread commercial introduction of SiPM-based whole-body TOF-PET systems (third generation TOF-PET systems) from all major manufacturers [22]–[26]. These new scanners achieve TOF resolutions varying from 214 ps FWHM [24] to 382 ps FWHM [27] depending, among others, on the properties of the crystals and SiPMs used, the degree of light sharing (number of crystals per SiPM), and percentage of crystal area covered by the SiPM array. The compact detector design achieved with SiPMs also allows for highly modular systems with variable axial length [27]. Additionally, the small size of SiPMs has allowed some of the vendors to improve the detector spatial resolution by using crystals that are less than 4 mm wide [24], [25].

In subsequent sections of this article, we broadly focus on: factors affecting time resolution, utilization of TOF information for improved image reconstruction, and quantifying the benefits of TOF imaging. Finally, we provide our perspective on the future prospects of TOF-PET, in particular where new detector advancements are leading us to, their impact on generating accurate PET images, and, what role TOF-PET has toplay in the latest advancements toward long axial FOV (AFOV) PET systems.

## II. Time Resolution

The TOF resolution of modern PET scanners is primarily determined by the timing performance of the scintillation detectors. An elaborate review of TOF-PET detector technology and the factors that affect time resolution has recently been published [28]. Here, we briefly review the theory of time resolution, relevant innovations in scintillation materials and photosensor technology, and the way in which time resolution is influenced by the design of the detector.

### A. Timing Theory

**Fig. 2.**
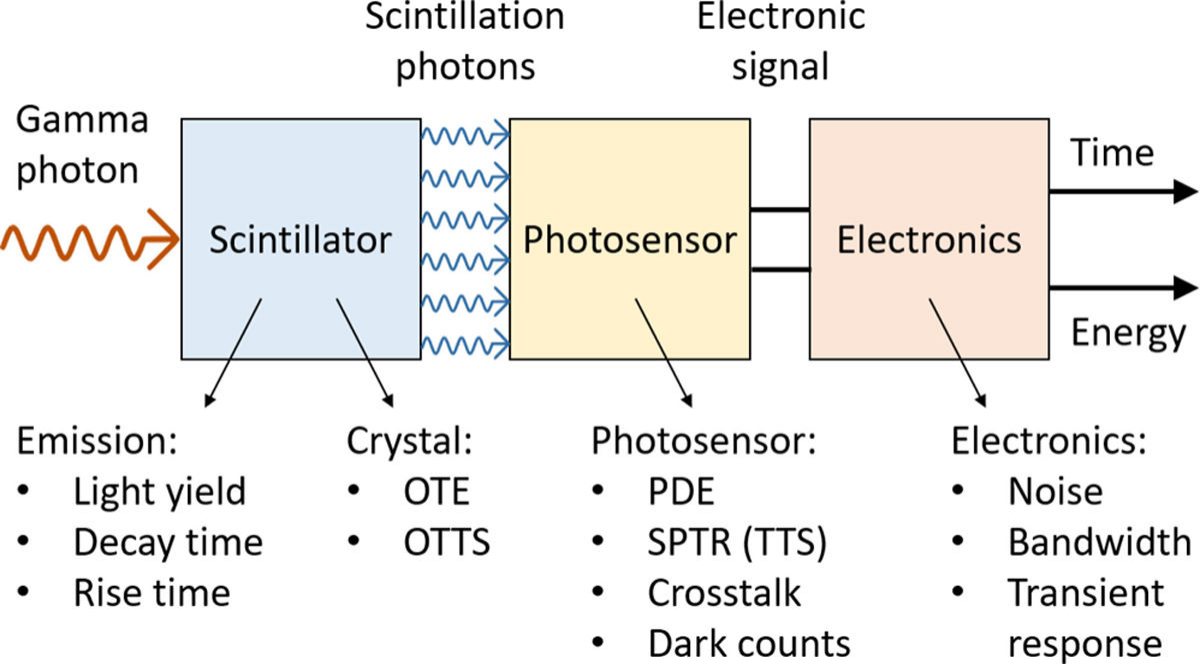
Schematic representation of a scintillation detector and some of the main factors influencing its time resolution. See text for the definitions of the abbreviations and further explanation.

Fig. 2 shows a schematic representation of a scintillation detector and some of the main factors that influence its time resolution. The absorption of a gamma photon with energy *E*_*γ*_ at a certain time Θ results in the emission of a number of scintillation photons *N*_*e*_ = *E*_*γ*_ *Y* (typically on the order of 10^4^), with *Y* the light yield of the scintillator. The optical transfer efficiency (OTE) determines the fraction of the emitted photons that will arrive at the photosensor. The photodetection efficiency (PDE) equals the fraction of the arrived photons that will finally be detected. The temporal distribution *p*_*td*_ (*t*|Θ) of the *N*_*d*_ detected photons is given by the convolution of three probability density functions: 1) the shape of the scintillation light pulse (often characterized by exponential rise and decay time constants, τ_rise_ and τ_decay_, respectively); 2) the optical transfer time spread (OTTS), which results from the transport of the scintillation photons within the crystal; and 3) the single-photon time resolution (SPTR) of the photosensor, which determines the uncertainty with which the arrival time of a single photon at the sensor can be measured. In the case of a PMT, the SPTR is also called the TTS. Other factors that may affect the time resolution include crosstalk, dark counts, noise, and the bandwidth and transient response of the readout electronics.

Hyman *et al.* developed a commonly used model of the time resolution of PMT-based scintillation detectors [29], [30]. They took into account *τ*_rise_ and *τ*_decay_, as well as the amplitude and shape of the single-photoelectron signal (SER), the TTS, and the gain dispersion *r*_*a*_ of the PMT. The result is commonly expressed in terms of the so-called Hyman function *H*(*τ*_rise_, *τ*_decay_, *σ*_SER_, *σ*_TTS_, *h*). Here, *σ*_SER_ and *σ*_TTS_ are the standard deviations of the SER and TTS, respectively, both of which are assumed to have a Gaussian shape. The parameter *h* is the trigger threshold as a fraction of the total pulse height. The standard deviation of the (Gaussian distributed) estimate of the time of interaction can then be written as

(1) $$\sigma_{t}=H\left(\tau_{\text{rise}},\tau_{\text{decay}},\sigma_{\text{SER}},\sigma_{\text{TTS}},h\right)\frac{r_{a}\tau_{\text{decay}\;}}{\sqrt{{\bar{N}}_{d}}}$$

with ${\bar{N}}_{d}$ the expected number of detected photons.

The Hyman model predicts the time resolution in the *infinitesimal-crystal approximation*, i.e., it ignores the OTTS. Cocchi and Rota showed that the OTTS cannot be neglected for crystal dimensions on the order of cm if the time resolution is in the range of a few hundred picoseconds [31]. Bengtson and Moszynski therefore added the OTTS to the Hyman model, under the assumption that the optical transfer times are Gaussian-distributed [32].

While the timing properties of scintillation detectors based on PMTs have been well understood for decades, SiPMs have fundamentally different characteristics and therefore require a new theory. Seifert *et al.* developed a probabilistic, and therefore a more generally applicable, model that can account for SiPM-specific properties, such as a highly asymmetric shape of the single-photon response and crosstalk, as well as electronic noise [33]. The model furthermore allows a more detailed modelling of the scintillation pulse, e.g., including multiple rise- and decay-time constants and a non-Poisson variance of *N*_*d*_.

Seifert *et al.* considered the detector output signal *v*_Σ_(*t*) as the sum of *N*_*d*_ single-photon signals *v*_*sps*_(*t*), all assumed to be statistically independent and identically distributed (IID) in time and amplitude. The onset of each *v*_*sps*_(*t*) is a random variable determined by: 1) the time of emission of the corresponding scintillation photon; 2) its optical transfer time; and 3) the SPTR of the photosensor. The photosensor gain spread and crosstalk are taken into account as stochastic processes influencing the formation of the *v*_sps_(*t*). The timing uncertainty can then be written as [33]

(2) $$\sigma_{t}\approx \frac{\sqrt{\frac{E\left[v_{\text{sps}}^{2}|{\bar{t}}_{\text{th}}\right]}{{\bar{N}}_{d}}+\frac{R_{\text{int}}^{2}}{2.35^{2}}E{\left[v_{\text{sps}}|{\bar{t}}_{\text{th}}\right]}^{2}+\frac{\sigma_{el}^{2}}{{\bar{N}}_{d}^{2}}}}{\frac{\partial }{\partial {\bar{t}}_{\text{th}}}E\left[v_{\text{sps}}|{\bar{t}}_{\text{th}}\right]}$$

where *E* is the (conditional) expectation operator, ${\bar{t}}_{\text{th}}$ is the time at which *E*[*v*_Σ_|*t*] crosses a given threshold value *V*_th_, *R*_int_ is the (FWHM) intrinsic energy resolution of the scintillator [34], and $\sigma_{el}^{2}$ is the electronic noise variance.

It should be noted that (2) reduces to (1) in the case where the scintillator and photosensor properties correspond to those assumed by Hyman *et al.* The equivalence of the Seifert and Hyman models for this special case is noteworthy as they were derived via conceptually different approaches. However, the Seifert model is considerably more versatile, not only because it allows more elaborate modeling of the scintillator and photosensor properties but also because it does not require the optical transfer, the photosensor single-photon timing performance, etc., to be described by Gaussian distributions.

Interestingly, the application of the aforementioned models shows that photon counting statistics form the dominant contribution to the time resolution of modern TOF-PET systems. As a result, Seifert *et al.* could show that the Cramér–Rao lower bound (CRLB) provides a useful measure of the time resolution achievable with a given detector [35]. In particular, for any unbiased estimator $\hat{\Theta }$ of the time of interaction

(3) $$\mathrm{Var}[\hat{\Theta }]\geq {\left[N_{d}\int_{-\infty }^{\infty }{\left(\frac{\partial }{\partial \Theta }p_{t_{d}}(t|\Theta )\right)}^{2}\frac{1}{p_{t_{d}}(t|\Theta )}dt\right]}^{-1}$$

where $p_{t_{d}}(t|\Theta )$ is the probability density of photon detection introduced at the beginning of this section.

Equation (3) is valid if the OTTS can be assumed to be constant, e.g., if the position of interaction of the gamma photon $\vec{x}$ within the crystal is the same in all events, or if the crystal can be considered infinitesimally small. In realistic TOF-PET detectors, the variation of $\vec{x}$ and the optical transfer can give rise to three causes of time resolution loss: 1) the dependence of the average optical transfer time (from $\vec{x}$ to the photosensor) on $\vec{x}$; 2) the OTTS for a given $\vec{x}$, as determined by the detector geometry and the properties of the optical interfaces; and 3) the variation of the OTTS with $\vec{x}$. Toussaint *et al.* [36], Loignon-Houle *et al.* [37] showed that the incorporation of these effects in the CRLB is nontrivial, but still arrived at a useful expression for the time resolution achievable with high-aspect-ratio crystals. It is possible to generalize Toussaint’s equation such that it also applies to other types of crystal, for example monolithic scintillator detectors [28].

**Fig. 3.**
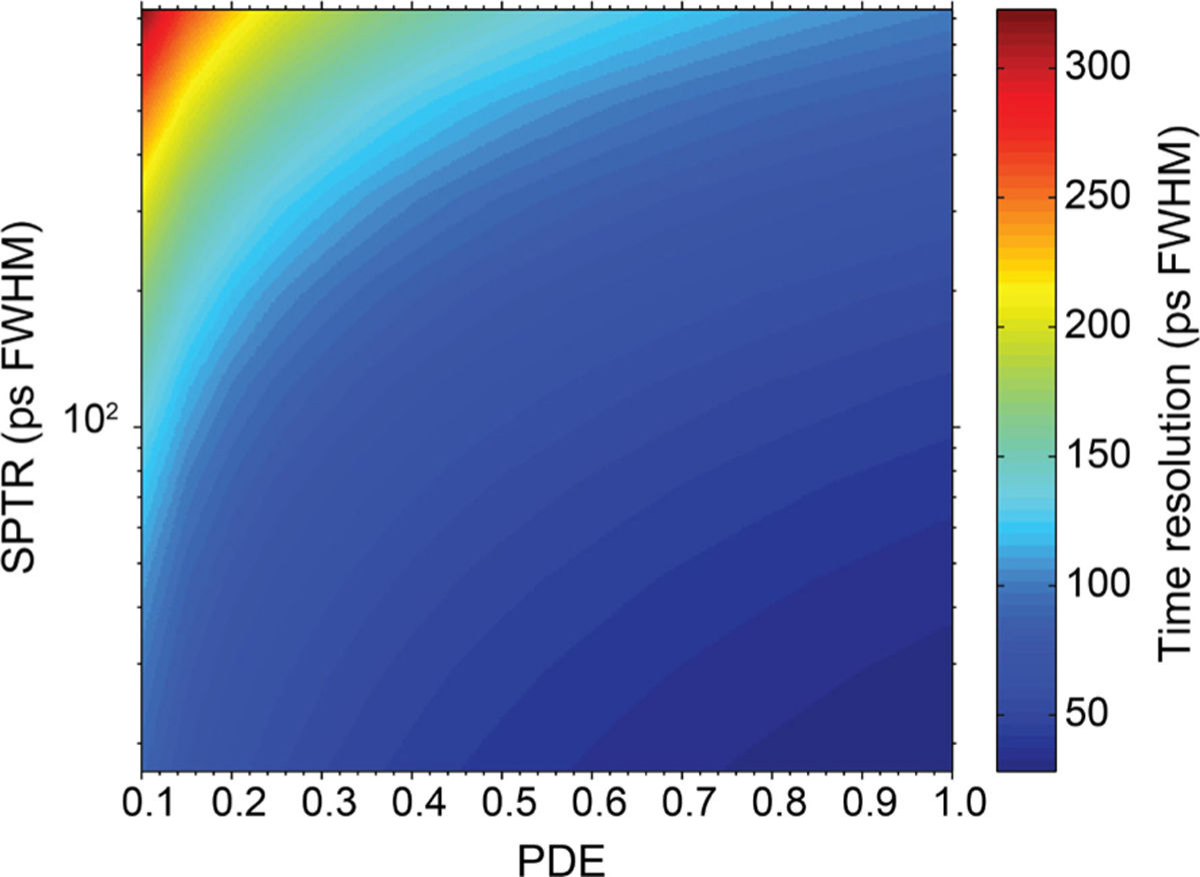
Cramér-Rao lower bound on the time resolution of two coincident detectors based on state-of-the-art Lu-based scintillation crystals (*Y* =33 ph/keV, *τ*_decay_ =33 ns, *τ*_rise_ =90 ps), as a function of the photosensors PDE and SPTR. This figure was originally presented in [38] and [39].

The CRLB quantifies the potential timing performance of a scintillation detector, independent of the time estimator used. The CRLB can be utilized, for example, to rationally optimize a hardware design and/or to calculate an objective reference against which the performance of a timing algorithm can be compared. Moreover, it can be used to explain and quantify general trends in scintillation detector timing performance. As an example, Fig. 3 shows the lower bound on the time resolution that can be achieved with a state-of-the-art Lu-based scintillator, as a function of the PDE and the SPTR of the photosensor.

It must be emphasized that the theory has been validated for detectors based on such fast and bright scintillators only. In particular, the application of CRLB theory to weak sources of prompt photon emission, such as done in e.g., [40]–[43], may yield overly optimistic results, as explained in more detail in [28].

In summary, the following general trends are observed in state-of-the-art TOF-PET detectors based on fast and bright scintillators, where photon counting statistics are the dominant contribution to the time resolution. First, the time resolution is inversely proportional to $\sqrt{{\bar{N}}_{d}}$ and, therefore, the square root of the scintillator light yield *Y*. Second, if *τ*_decay_ is larger than the *τ*_rise_, the OTTS, and the SPTR, as is commonly the case, the time resolution is also proportional to $\sqrt{\tau_{\text{decay}}}$. Thus, $\sqrt{Y/\tau_{\text{decay}}}$ is a useful figure of merit (FOM) for a TOF-PET scintillator (higher values of this FOM corresponding with better timing potential). The rise time becomes important only if it is larger than both the OTTS and the SPTR, in which case the time resolution also becomes proportional to $\sqrt{\tau_{\mathrm{rise}}}$. Third, the PDE and SPTR determine the time resolution that can be obtained with a given photosensor. Finally, a relatively small number of early detected photons often appear to carry most of the timing information. Yet, the lowest variance is often not associated with the very first photon detected [28].

### B. TOF-PET Scintillators

The development of scintillators for medical imaging is an active field of research [44]–[47]. Table I lists several scintillators that have been investigated for use in TOF-PET systems.

It is evident from timing theory (Section II-A) that a TOF-PET scintillator should have a short decay time as well as a high light yield. A high light yield also makes it easier to obtain signals with a high SNR from the detector, which is important to achieve good energy and spatial resolution. The optimization of the detector spatial resolution is furthermore facilitated by reducing the average path length of the annihilation quanta within the crystal until full absorption. The probability of photoelectric interaction per unit path length is proportional to $\rho Z_{\text{eff}\;}^{k}$, with *ρ* the density of the scintillator, *Z*_eff_ its effective atomic number, and *k* ≈ 3.5. For Compton interactions, this probability is roughly proportional to *ρ*. It follows that both *ρ* and *Z*_eff_ are important scintillator properties.

This is all the more so, because *ρ* and *Z*_eff_ also determine the detection efficiency *η*_det_ of the detector. As will be elaborated in Section IV, the benefit of image reconstruction utilizing a TOF resolution *t* can be understood as an improvement of variance (noise) by a factor proportional to *D*/Δ*t*, with *D* the diameter of the object imaged [17]. Thus, one may say that the effective sensitivity of a TOF-PET system is proportional to

(4) $$S_{\text{eff},D}\propto \eta_{\text{det}\;}^{2}\eta_{\text{geom}\;}\frac{D}{\Delta t}$$

with *η*_geom_ the geometrical efficiency (angular coverage) of the system. Note that *S*_eff,*D*_ goes as the square of *η*_det_, since a pair of annihilation quanta must be detected to obtain a valid PET event. Also note that the image SNR will be proportional to $\sqrt{S_{\text{eff},D}}$. It follows that the use of a detector with better time resolution but lower density, for example, does not necessarily result in better imaging performance.

The importance of this point is illustrated by the results obtained with the first generation of TOF-PET scanners based on BaF_2_ and CsF that were described earlier in Section I-B. The fast cross-luminescence in these materials enabled TOF imaging, but the low light yield and density led to inferior imaging performance compared to non-TOF systems based on the much denser scintillator BGO.

The discovery of the fast, bright, and dense scintillator LSO:Ce in the mid-1990’s [14] renewed the interest in TOF [16], [48], [49]. The first of the second-generation of clinical TOF-PET scanners [18] was based on a similar material, lutetium-yttrium oxyorthosilicate (LYSO:Ce), in which a small fraction of the lutetium ions is replaced by yttrium [50], [51].

Around 2000, Ce-doped lanthanum bromide (LaBr_3_:Ce) and cerium bromide (CeBr_3_) were found to have high light output, a short decay time, as well as excellent energy resolution [52], [53]. TOF resolutions better than 100 ps FWHM were reached for the first time using LaBr_3_:Ce crystals coupled to PMTs [54] as well as SiPMs [55]. Interestingly, the scintillation rise time was found to increase with decreasing Ce concentration. Commercially grown LaBr_3_:Ce with a Ce concentration of 5% has a rise time of several hundreds of ps, sufficiently long that it significantly affects the time resolution of LaBr_3_:Ce based detectors [56], [57].

A whole-body TOF-PET scanner was built using LaBr_3_:Ce crystals [58], achieving a system TOF resolution of 375 ps FWHM. Also, the 7% FWHM energy resolution helped to improve scatter correction. However, the relatively low *ρ* and *Z*_eff_ led to increased intercrystal scattering and a reduced detection efficiency compared to L(Y)SO:Ce.

Today, essentially all clinical TOF-PET systems utilize LSO:Ce or LYSO:Ce. The crystal growth process has been optimized over time, and crystals with excellent and uniform properties are readily available [41], [56], [59]–[66]. It appears that co-doping of these materials with divalent ions, Ca^2+^ in particular [65], [67], [68], allows for a substantial improvement of $\sqrt{Y/\tau_{\text{decay}}}$ and, therefore, the achievable time resolution [40], [69], [70]. Excellent timing has also been demonstrated with so-called lutetium fine silicate (LFS) [71]–[74]. Another promising material is lutetium-gadolinium oxyorthosilicate (LGSO:Ce). It appears that its light yield and decay time can be controlled by varying the Ce concentration, which led to the development of so-called LGSO-Fast [74]–[76]. Results, such as these show that careful optimization of the material composition, co-doping, crystal growth process, etc., (approaches sometimes referred to as *scintillator engineering*), may allow for a substantial improvement of a materials’ timing potential.

### C. TOF-PET Photosensors

The readout of scintillators in TOF-PET detectors requires photosensors with internal gain [77], capable of detecting single photons with high PDE and SPTR. PMTs have been the device of choice since the early days of PET. Their principle of operation [78] and timing properties (Section II-A) are well understood. The PDE is primarily determined by the quantum efficiency (QE) of the photocathode. PMTs typically have a QE of ~25% around 400 nm, although some photocathodes reach a QE of up to ~40% [79], [80]. The SPTR of a PMT is often referred to as TTS, as it is primarily determined by the spread in the transit times of the photoelectrons between the photocathode and the first dynode. PMTs optimized for fast timing applications may have a TTS better than ~200 ps FWHM [54], [81]–[84], while so-called microchannel-plate (MCP) PMTs may have even better TTS values [85]–[87]. The high internal gain (~10^6^–10^8^), low dark current, and low capacitance (~10 pF) of PMTs impose relatively mild requirements on the readout electronics. As mentioned in Section I-B, a variety of PMT-based TOF-PET systems have been brought onto the market, offering time resolutions in the range of 450–600 ps FWHM. Moreover, several PMT-based prototype whole-body systems with time resolutions between 300–400 ps FWHM have been developed [58], [88], [89].

**Fig. 4.**
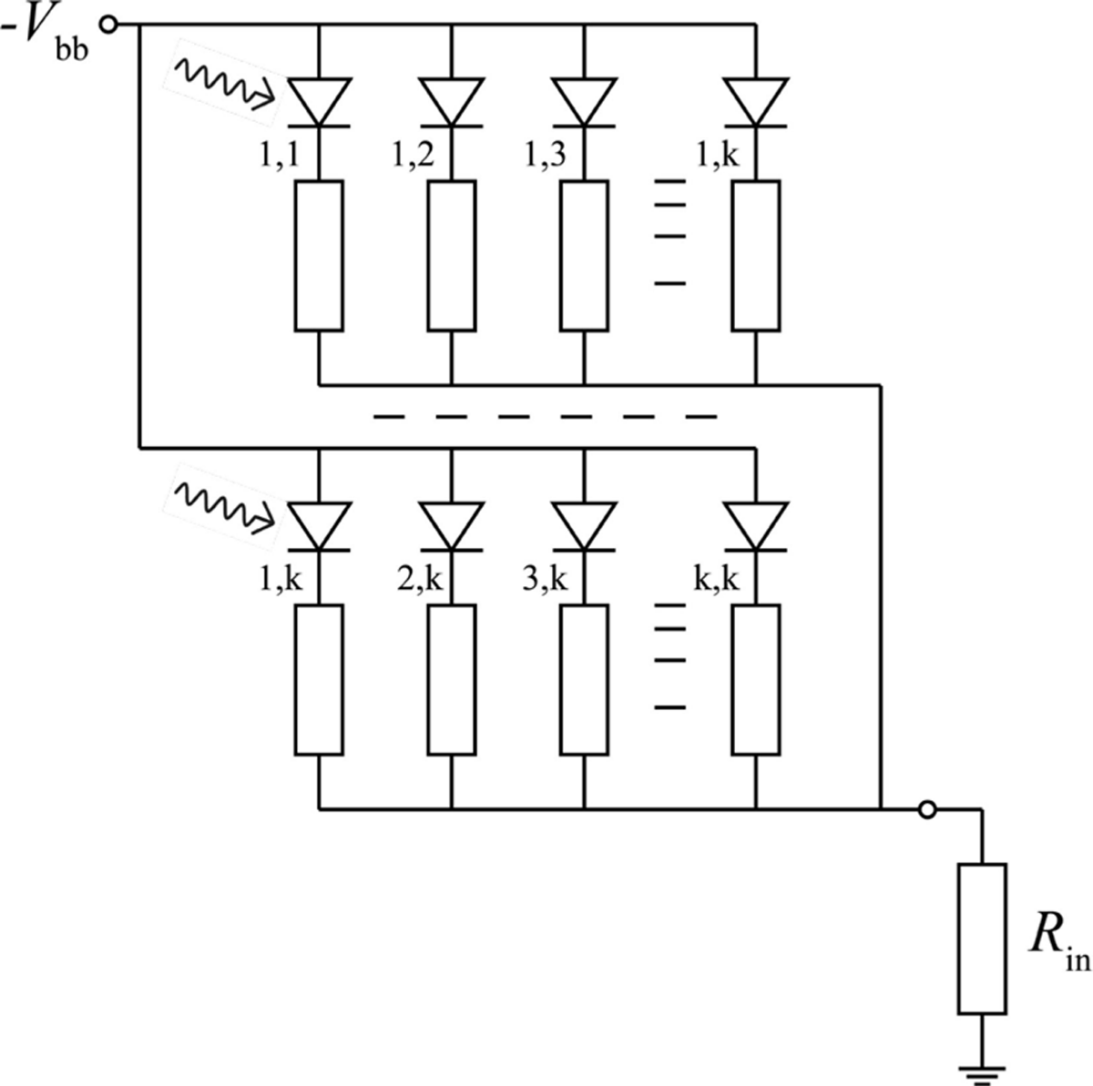
Parallel electrical connection of many SPADs in a silicon photomultiplier; the symbol *V*_bb_ denotes the bias voltage and *R*_in_ is the input resistanceofthereadoutcircuit.Thisfigurewasoriginallypublishedin [98] (©2020Springer).

Compared to PMTs, photosensors based on semiconductors have several advantages, such as a potentially higher PDE, small size, low-voltage operation, flexibility in geometric design, ruggedness, and unperturbed performance in magnetic fields (enabling MRI-compatibility). With the invention of the single-photon avalanche diode (SPAD), a solid-state single-photon detector with high internal gain (10^5^–10^7^) became available. SPADs are photodiodes operated in Geiger mode; the detection of a photon triggers a self-quenched discharge that produces a fixed amount of charge. By connecting a large number (typically 10^2^–10^5^) of SPADS in parallel on a monolithic CMOS device, as shown schematically in Fig. 4, a proportional photosensor can be realized: the SiPM [90]–[94].

In practice, several phenomena limit the proportionality of SiPMs. These include saturation (which may occur if SPADs are illuminated by more than one photon within a brief time interval), after-pulsing (generated by trapped charge carriers released some time after the original pulse), and crosstalk (discharges triggered in neighboring SPADs by photons produced in the initial avalanche). These effects need to be taken into account to fully understand the response of SiPM-based detectors [95]–[97].

The PDE of a SiPM is commonly described as the product of its fill factor (the sum of the SPAD active areas divided by the total device area), the SPAD QE (the probability that a photon creates an electron-hole pair), and the trigger probability (the probability that the electron-hole pair triggers an avalanche). SiPMs with PDEs of up to 60% at 420 nm are currently available [99].

The SPTR of a SiPM is determined primarily by the SPTR of its SPADs. Additional contributing factors include the SPAD gain spread, variation in pulse shape and pulse propagation delay due to different metal trace lengths between the SPADs and the SiPM output pad, the cumulative dark count rate, and unfavorable shaping of signals due to SiPM parasitic impedances. State-of-the-art SiPMs have SPTR values in the range of 50–150 ps FWHM [100].

**Fig. 5.**
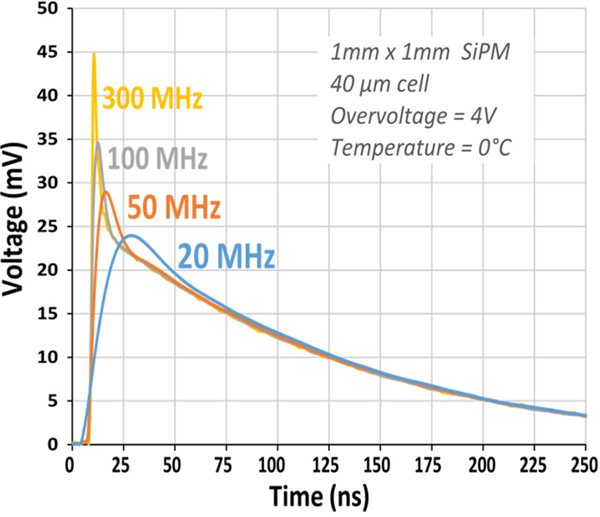
Influence of readout electronics bandwidth on the single-SPAD response of a 1×1 mm^2^ SiPM (FBK NUV-HD 2018) with 40 *μ*m SPAD pitch. The fast component is clearly visible at high bandwidth. This figure was originally published in [102].

As mentioned in Section I-B, the most recent TOF-PET scanners of essentially all commercial manufacturers are equipped with SiPMs. Some of these systems have a time resolution approaching 200 ps FWHM, which is largely due to the excellent PDE and SPTR of SiPMs. The electronic properties of SiPMs, on the other hand, are somewhat less favorable than those of PMTs. They have a relatively high capacitance, for example. Moreover, the single-SPAD signal (SSR), i.e., the signal observed when a single SPAD fires, exhibits a fast rise time (<<1 ns), followed by an exponential decay that results from the recharging of the SPAD. The recharge time constant is typically on the order of tens of ns [101], much larger than the fall time of a few ns of a PMT. In addition to this so-called “slow” component, some SiPMs exhibit a rapid initial decay, commonly called the “fast” component of the SSR (see Fig. 5).

Obviously, the use of SiPMs with a short recharge time and a prominent fast component facilitates good timing in TOF-PET detectors. To maintain a favorable pulse shape, the readout electronics must have sufficient bandwidth, as well as the lowest possible input impedance at signal frequencies [103], [104]. Still, the rising slope of a SiPM-based scintillation detector pulse will be significantly smaller than that of a PMT-based detector with equal gain, even if the scintillation pulse and the SSR both have a short rise time. This is because the detector output pulse equals the convolution of these two functions, the shape of which is determined primarily by the scintillation decay time and the SiPM recharge time (a more complete explanation including examples can be found in [28]). One of the consequences of this is that the timing performance of a SiPM-based detector may be more sensitive to electronic noise. Moreover, the long tails of SiPM dark counts (and the associated crosstalk [105]) give rise to low-frequency noise, so the readout electronics should preferably provide for some form of baseline restoration [55], [106]–[109]. Indeed, SiPM readout is a topic of active research and the many innovations in this field have contributed significantly to the excellent timing obtained with SiPM-based detectors today [100], [104], [107], [110]–[112].

**Fig. 6.**
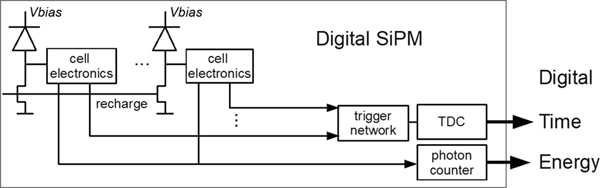
Schematic representation of a dSiPM. This figure was originally published in [115] (©2009 IEEE).

A different approach to solve the readout challenges associated with SiPMs is to integrate digital circuitry for data acquisition and device control into the sensor chip itself. Such devices are called digital SiPMs (dSiPMs). The logic circuit integrated locally with each SPAD (Fig. 6) executes a quenching and recharge cycle when it detects a discharge and sends a trigger signal to the onboard photon-counting and time-to-digital conversion (TDC) electronics.

The local detection of a discharge in a dSiPM makes the time pickoff less sensitive to unfavorable pulse shaping, SPAD gain variation, and dark counts. On the other hand, factors, such as skews in the digital trigger network, clock distribution jitter, and TDC resolution and nonlinearity may affect the timing performance of a dSiPM [113], [114]. Furthermore, the addition of logic circuitry on the sensor may go at the expense of fill factor and, therefore, PDE.

Frach *et al.* [115], [116] developed the first dSiPM specifically for PET, known today as the Philips digital photon counter (DPC). The PDE of this device exceeds 40% at 420 nm and the SPTR of the SPADs, single pixels and the full sensor chip were found to be ~48 ps FWHM, ~100 ps FWHM, and ~170 ps FWHM, respectively [117], [118].

The DPC is currently the only dSiPM being used in a commercially available TOF-PET system [22], [119]. However, other types of dSiPM are under development, e.g., [120]–[122]. In particular, the development of 3D-integrated dSiPMs offers an interesting path to resolve the tradeoff between PDE and SPTR that imposes compromises in the design of 2D dSiPMs [123], [124].

### D. Optimization of Scintillation Detector Design

The use of scintillators with optimized timing performance and SiPMs with high PDE and SPTR both contribute to the excellent time resolution offered by recent TOF-PET scanners. Another factor not to be overlooked is the reduction in OTTS that has become possible due to the introduction of SiPMs. Section II-A discussed the three causes of time resolution loss that occur in noninfinitesimal scintillation crystals [28], [36]. Each of these effects are minimized when as many of the scintillation photons as possible are transferred to the photosensor as quickly as possible. This is difficult to achieve in typical PMT-based PET detectors, in which a large number of crystals (on the order of ~10^2^) share their light over a 2 × 2 PMT array spanning an area of some ~25 cm^2^. In comparison, the degree of light-sharing in SiPM-based TOF-PET designs is considerably reduced. It is even possible to utilize a one-to-one coupling geometry, in which each crystal is read out by its own SiPM [119], [125]. The careful application of high-quality reflectors and optical glues in the assembly of the detector further contributes to the excellent timing performance of modern TOF-PET detectors.

Despite the recent advances in detector design, the influence of the OTTS has not been fully eliminated and remains a bottleneck for the development of systems with sub-100 ps TOF resolution. Thus, the development of methods to further minimize and/or actively correct events for this effect is an emerging field of research. Some approaches in this direction are discussed in Section V-A.

## III. Utilization of Time Resolution in PET Image Reconstruction

### A. TOF-PET Reconstruction Basics

As early as in the 1980s, with the development of the first generation of TOF-PET scanners, the classical analytical *filtered back-projection* algorithm (FBP) was extended for reconstruction of 2D TOF sinograms. Because TOF-PET data are redundant, the filter in the radial direction applied before TOF back-projection is not uniquely determined by the problem. Instead, different combinations of back-projection weights (TOF kernels applied during back-projection) and radial sinogram filters can be applied for reconstruction. An extreme example is to ignore the TOF information during back-projection, which produces the conventional non-TOF FBP algorithm. The other extreme is to back-project the data by assigning each event to the most likely annihilation point. Snyder *et al.* [4] and Tomitani [6] developed a TOF FBP-like algorithm for 2D PET reconstruction. Tomitani showed that for minimal variance in the center of a uniform cylinder, a “confidence-weighted” back-projection should be used in this algorithm. This means that during back-projection, the TOF-PET data are smoothed with a Gaussian TOF kernel that models exactly the TOF uncertainty in the direction of the LOR. The corresponding reconstruction filter in the frequency domain, to be applied in the radial direction before the back-projection, is the convolution of the ramp filter and a Gaussian

(5) $$h(v)=4\pi^{2}\sigma^{2}\int_{-\infty }^{\infty }du|u|e^{-4\pi^{2}\sigma^{2}{\left(u-v\right)}^{2}}$$

where *σ* is the standard deviation of the TOF-kernel. Consequently, the corresponding filter kernel in the spatial domain is simply the product of the conventional ramp filter kernel *h*_ramp_(*x*) with a Gaussian with standard deviation $\sqrt{2}\sigma$

(6) $$h(x)=h_{\mathrm{ramp}}(x)\sqrt{4\pi }\sigma e^{-\frac{x^{2}}{4\sigma^{2}.}}$$

A comparison of several TOF FBP reconstruction kernels by Watson [126] confirmed that confidence-weighted back-projection produces low-variance images, if the object is large compared to the TOF resolution.

**Fig. 7.**
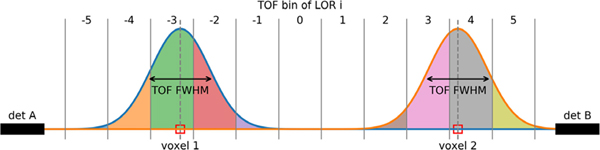
Illustration of the TOF-PET forward model for two voxels containing activity on an LOR divided into 11 TOF bins. The contribution of a given voxel to a TOF bin is given by the integral of a Gaussian kernel centered on the voxel. Note that every voxel only contributes to a few TOF bins (a smaller part of the LOR) and that any TOF bin only receives contributions from one of the two voxels if the TOF FWHM is much smaller than the distance between the voxels.

In current 3D *iterative PET image reconstruction* algorithms, the additional information provided by TOF is usually incorporated within the forward model by subdividing the LORs into smaller TOF bins as illustrated in Fig. 7. The spatial width of those TOF bins should be substantially smaller than the blurring caused by the TOF resolution of the system to allow for sufficient sampling. In the discretized setting, the contribution of a voxel *j* containing an activity concentration *λ*_*j*_ to the TOF bin *t* along geometrical LOR *i*, can be described as

(7) $${\bar{y}}_{it}=\sum_{j}n_{i}g_{itj}k_{ij}\lambda_{j}+s_{it}=\sum_{j}c_{ijt}\lambda_{j}+s_{it}$$

where *k*_*ij*_ are geometrical projection weights, *n*_*i*_ are multiplicative corrections, such as normalization and attenuation and *s*_*it*_ are additive contaminations, such as randoms and scatter. The TOF kernel *g*_*itj*_ is the sensitivity of TOF bin *t* along LOR *i* to activity at voxel *j*. It represents the blurring along LOR *i* caused by the finite TOF resolution. It is usually modeled as a Gaussian function of the distance between voxel *j* and the point corresponding to bin *t* along LOR *i* (see Fig. 7). To also account for the effect of the bin width, that Gaussian can be convolved with a rectangular kernel representing the bin sensitivity profile. In contrast to the non-TOF forward model, we see that every voxel contributes only to a smaller part of the geometrical LOR (a few TOF bins), and that measured data in a single TOF bin can only originate from a smaller subregion of the LOR. Including this forward model into an (ordered-subset) maximum-likelihood expectation–maximization (ML-EM) algorithm leads to the well-known ML-EM update for TOF-PET [127]–[129], given by

(8) $$\lambda_{j}^{n+1}=\frac{\lambda_{j}^{n}}{\sum_{i}\sum_{t}c_{ijt}}\sum_{i}\sum_{t}c_{ijt}\frac{y_{it}}{{\bar{y}}_{it}\left(\lambda^{n}\right)}.$$

As discussed in more detail in Section IV, the use of TOF information in the (iterative) reconstruction process has several advantages:

1. reduction of variance (SNR gain) in objects that are bigger than the TOF FWHM;
2. faster convergence to the maximum-likelihood solution;
3. more uniform convergence.

Note that compared to non-TOF systems, the reconstruction problem in systems with sufficient TOF resolution becomes “local.” That is, regions in the object that are sufficiently far apart from each other do not contribute to the same data bins. This, in turn, means that the signal from a small object measured in one TOF bin on a given LOR is “contaminated” less by events emitted from surrounding (background) activity along the same LOR. The resulting SNR increase in the acquired data is propagated into the reconstruction as shown in [6] and [130]. Moreover, TOF-MLEM is more robust in the presence of inconsistent data (e.g., due to local errors in the attenuation image) as shown in [131].

Compared to non-TOF MLEM, the TOF-MLEM update is computationally more complex since the TOF kernels and the additional sums over the TOF bins have to computed in the forward- and back-projections. Due to the size of the system matrix, this is commonly performed “on-the-fly.” The memory requirement in a traditional sinogram MLEM update increases linearly with the number of used TOF bins. Consequently, sinogram-based TOF-MLEM without data rebinning becomes more and more computationally demanding with improving (smaller) TOF resolution. In current whole-body scanners with an axial FOV of 20–25 cm and a TOF resolution of 200–400 ps FWHM (3–6 cm FWHM), full TOF sinograms are already very sparse, naturally favoring a *list-mode reconstruction* approach [132], [133]. However, note that improved TOF resolution also allows for more aggressive sinogram rebinning that can help to reduce the computational burden [134]–[137].

As shown in [138], the use of TOF kernel widths that differ from the true system kernel width leads to artifacts in the reconstruction—especially in uniform regions. This underlines that precise knowledge of the TOF kernels is crucial for accurate TOF-MLEM reconstructions. Fortunately, data-driven ML techniques can be used to estimate a global TOF kernel width [138] or even an LOR-dependent correction factor for the TOF kernel width [139] in case the TOF resolution is LOR-dependent.

Another additional complexity of TOF-MLEM is the fact that additive scatter contaminations become TOF-bin dependent such that more advanced and complex methods for scatter estimation have to be used [140], [141].

### B. Advanced Reconstruction Methods Enabled by TOF

In emission and transmission tomography, there is almost always some redundancy in the projection data. For ideal data, the redundant part of the data should be compatible with the rest of the data. This requirement can be explicitly formulated in so-called consistency conditions. If the system is modelled correctly during reconstruction, then only the noise creates some violation of the consistency conditions. The availability of TOF information makes the data inherently richer, increasing their redundancy and therefore imposing new consistency conditions [135], [136], [142]. This extra information can be exploited to estimate additional parameters. For example, it has been proposed to utilize the TOF information to reconstruct scattered coincidences [143], [144], to estimate the attenuation sinogram or the attenuation image from the PET emission data [145], or to reconstruct data from systems with limited angular coverage [146], [147]. Here, we focus on the latter two applications, which we consider to have potential for significant impact on PET imaging.

#### 1) Joint Estimation of Emission and Attenuation:

In 2012, Defrise *et al.* [145] proved that due to consistency conditions, 2D TOF-PET data determine the radial and angular derivative of the forward-projected attenuation image such that the attenuation sinogram is determined up to a constant. This also holds true for fully 3D-TOF-PET, except that there might exist multiple constants in cases where the object contains nonsimply connected regions.

Defrise *et al.* also proposed a simple analytical algorithm to estimate the gradient of the forward-projected attenuation image. A variance analysis of this algorithm for a simplified object with centered Gaussian activity distribution revealed that the variance of the estimated gradient is proportional to

(9) $$\frac{{\left({\text{FWHM}}_{\text{object}}^{2}+{\text{FWHM}}_{\text{TOF}}^{2}\right)}^{\frac{3}{2}}}{{\text{FWHM}}_{\text{object}}^{4}}.$$

This clearly demonstrates that improving the TOF resolution reduces the variance of the attenuation estimate in joint estimation.

**Fig. 8.**
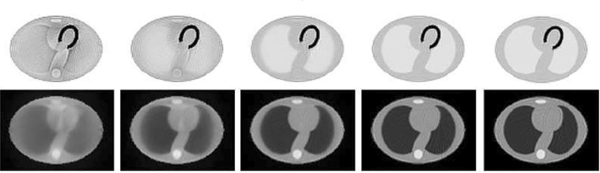
Noise-free simulation of joint estimation of activity (top) and attenuation image (bottom) with MLAA for a 2D thorax phantom for different TOF resolutions [40 cm/2667 ps (non-TOF), 20 cm/1333 ps, 10 cm/667 ps, 5 cm/333 ps, 2.5 cm/167 ps from left to right]. All reconstructions used 50 iterations and 32 subsets. This figure was originally published in [148] (©2012 IEEE).

Rezaei *et al.* [148] proposed an iterative algorithm to jointly estimate the activity and attenuation images (MLAA). The authors demonstrated that the availability of TOF information removed the crosstalk between activity and attenuation in the iterative estimation, which is usually very prominent when using non-TOF data as shown in Fig. 8.

Improved TOF resolution also leads to a faster convergence of joint estimation algorithms. Nuyts *et al.* [149] and Defrise *et al.* [150] also proposed and analyzed an iterative algorithm to jointly estimate the activity image and the attenuation sinogram directly (MLACF). Moreover, Rezaei *et al.* [151] proposed another iterative algorithm to estimate nonrigid deformation fields to correct for mismatches between the emission and attenuation image, e.g., due to respiratory motion (MLRR). Many other groups have been working on this problem and a recent overview is given by Berker and Li [152].

Very recently, different research groups have shown promising results with MLAA-like algorithms on different clinical data sets—see [153]–[156]. A limitation of all these joint estimation algorithms is the “missing-scale problem” caused by the fact that the attenuation sinogram is only determined up to a constant. This problem is usually solved by, including prior information, e.g., regions with known attenuation coefficients from a different modality, or by scaling the total activity of the reconstruction.

An attractive feature of MLAA is that it generates directly the attenuation image, which is needed for scatter correction and for determining the scale. If prior knowledge about the attenuation image is available, incorporating it in MLAA is straightforward. MLACF estimates instead the attenuation sinogram, which makes it difficult, if not impossible, to incorporate such prior knowledge. However, because estimating the sinogram requires fewer computations and converges faster, MLACF is faster than MLAA. MLACF does not impose consistency to the attenuation sinogram, it estimates an effective sensitivity for every LOR. As a result, it automatically corrects for residual normalization errors.

Recently, deep learning techniques (e.g., convolutional neural networks) have been used to improve the quality of the attenuation images obtained from MLAA [157], [158]. Also, it was recently shown [139] that joint estimation of activity and attenuation is more sensitive to inaccuracies in the TOF kernel, such as the exact TOF resolution and possible coincidence timing offsets, indicating that the required precision in the TOF calibration and modeling needs to be improved for future systems with even better TOF resolution.

#### 2) Reconstruction of Limited-Angle TOF-PET Data:

PET systems with limited angular coverage have gained the interest of different research groups. Examples of limited-angle PET systems are breast scanners with integrated biopsy solutions and dedicated heart and prostate systems [159]. Other examples are helmet-type PET scanners for brain [160] and dual-panel systems for *in-vivo* dosimetry in particle therapy [161]–[165]. Unfortunately, reconstruction of non-TOF PET data with limited angular coverage (i.e., PET systems for which the local Tuy condition is not satisfied for all voxels in the FOV) suffer from strong artifacts, such as strong blurring in one direction [166], [167]. Crespo *et al.* [146] and Surti and Karp [147] initially demonstrated in simulation studies of in-beam PET and dedicated breast scanners with varying angular coverage, that the use of TOF information strongly reduces the limited-angle artifacts observed in the non-TOF reconstructions. This idea was subsequently demonstrated experimentally in measurements performed on a clinical [168] and a proto-type [89] whole-body PET scanner, as well as benchtop imaging systems for proton therapy dose verification [162], [169].

Li *et al.* [142] showed that TOF information decreases the area in Fourier space that has no frequency information (shadow zone) for the object due to limited-angle data collected in a dual panel PET scanner. The authors concluded that: “… improving TOF time resolution, can … shrink the shadow zones. TOF measurement with currently achievable time resolution can reduce, but cannot eliminate, these artifacts.” As TOF resolution continues to improve, these shadow zones will get smaller and smaller and the amount of artifacts in the reconstructions will decrease. In the hypothetical case of “perfect TOF resolution” (or a TOF blurring that is smaller or equal to the detector spatial blurring), reconstruction of TOF-PET data from a two-plate system would become possible without any limited-angle artifacts.

**Fig. 9.**
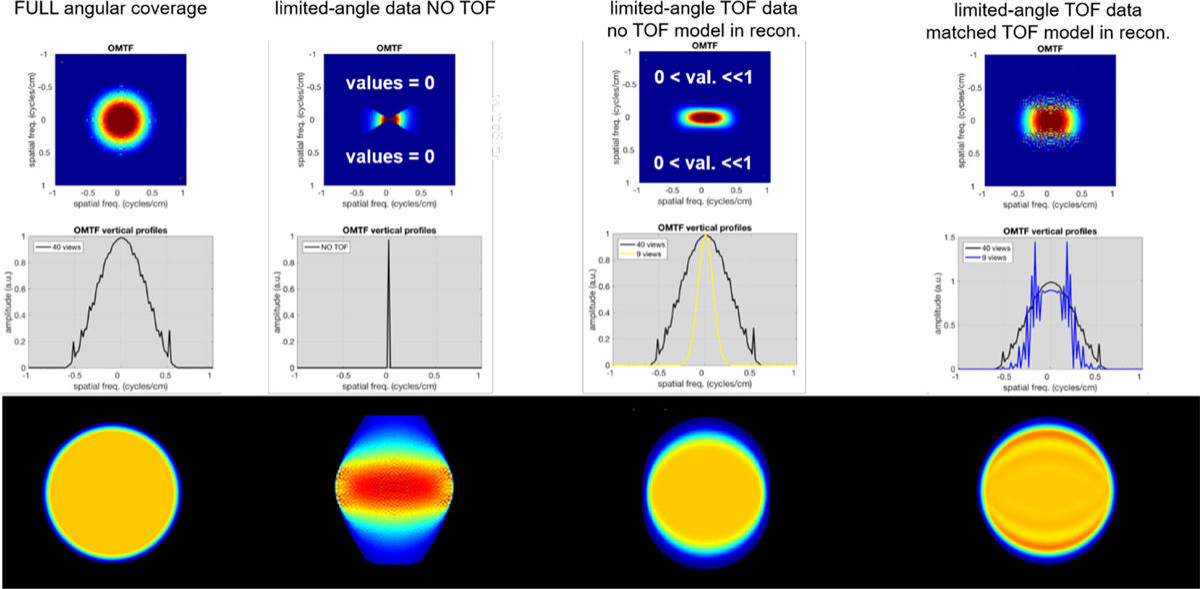
Illustrations of reconstructed images and their spectral content for the full angular coverage and limited-angle systems. Top row: Object-specific modulation transfer functions (OMTF, [2]) of the reconstructed images slightly smoothed to focus on the shape. Middle row: Central vertical profiles through the OMTFs. Bottom row: Corresponding reconstructed images. 1^st^ column: Full angular coverage reconstruction (the small ripples are due to discretization). 2^nd^ column: Limited-angle reconstruction from non-TOF data (i.e., for data acquired on a system without TOF capabilities). 3^rd^ column: Limited-angle reconstruction from TOF data, but without TOF modeling (i.e., for data acquired on a system with TOF capabilities, but reconstructed without modeling the TOF uncertainty). 4^th^ column: Limited-angle TOF reconstruction using matched TOF kernel. This figure was originally published in [170] (©2020 IEEE).

Recently, Gravel *et al.* [170] showed that iterative TOF reconstructions from limited-angle data using a matched TOF kernel suffer from ringing artifacts similar to Gibbs artifacts caused by point-spread function modeling. These artifacts, however, can be mitigated using different regularization approaches. The authors also visualized the object-specific modulation transfer function (OMTF) for non-TOF and TOF reconstructions, as shown in Fig. 9. Even without modeling of the finite TOF blurring in the reconstruction, TOF information helps to partly recover missing parts of the OMTF. When using a matched, finite TOF blurring in the reconstruction, a bigger part of the OMTF can be recovered, however certain frequency bands are overamplified.

Vergara *et al.* [171] showed in a simulation study using a rescaled MLACF algorithm that joined estimation of activity and attenuation is also possible for limited-angle two-plate PET system, which potentially allows for quantitative imaging with those systems.

## IV. Quantification of TOF Benefit

The earliest attempts to quantify the benefit of TOF-PET were made in the 1980s, where it was estimated that the reduced propagation of noise during forward- and back-projection leads to a gain in SNR given by √*D*/Δ*x*, where *D* is the size of the object being imaged [4], [5]. The Poisson nature of PET data allows one to equate this SNR gain into an effective sensitivity gain of *D*/Δ*x* that forms the first-pass estimate of any gains in TOF-PET [e.g., (4)]. However, this derivation has some limitations: it assumes a rectangular TOF kernel as opposed to the more realistic Gaussian kernel, it does not account for random coincidences in the data, it assumes an analytic reconstruction algorithm, and it is a measure of SNR gain at the center of a uniform cylindrical activity distribution.

The effect of the Gaussian TOF kernel and some post-reconstruction filtering was considered by Tomitani [6] in deriving an estimate of the TOF sensitivity gain of *D*/(1.6*x*) that was subsequently verified via measurements [128], [172]. This predicted gain has also been confirmed for OSEM reconstructions from PET simulations [130]. An alternative derivation, independent of the reconstruction algorithm, is given in the Appendix.

**Fig. 10.**
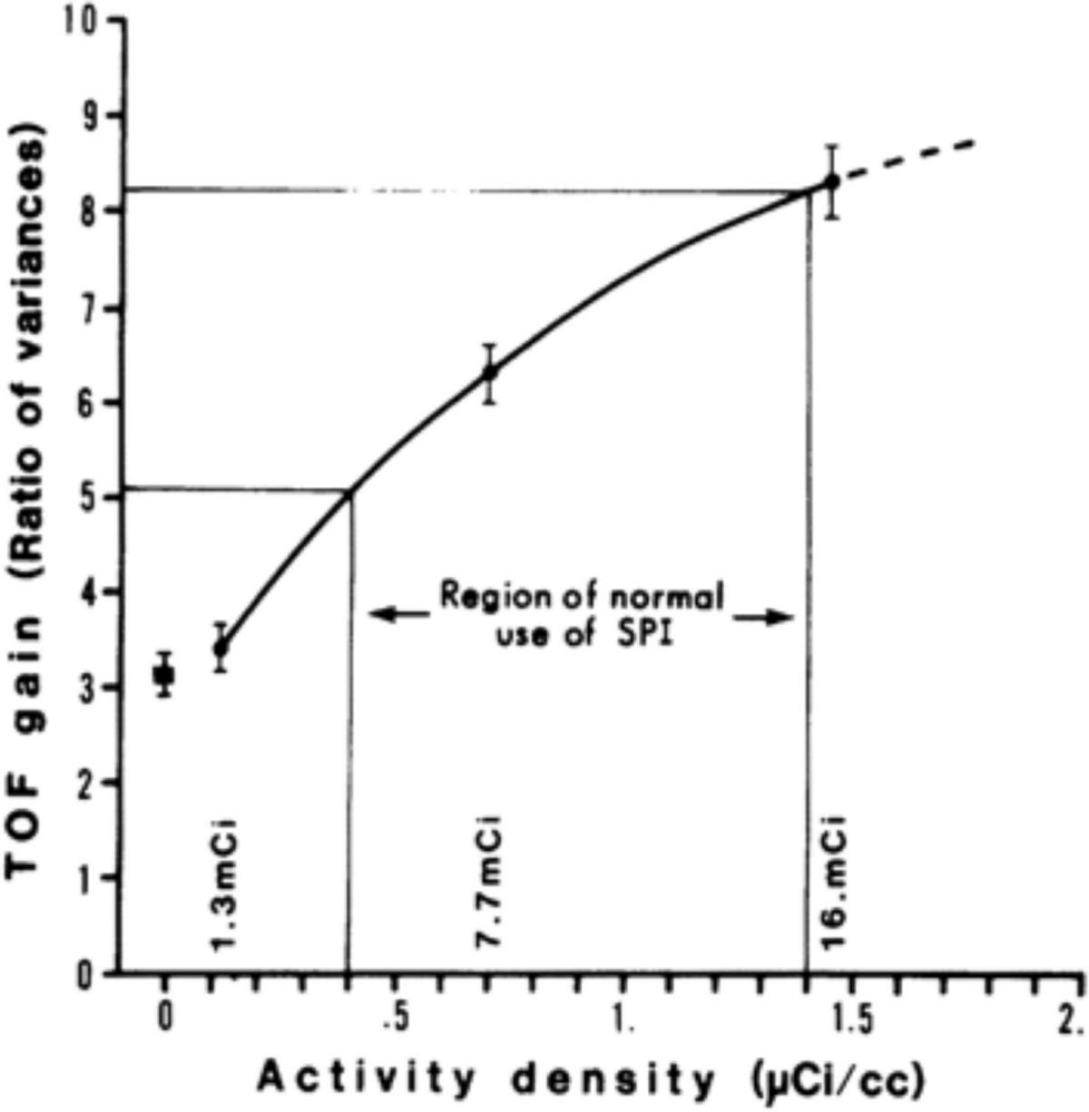
TOF gain relative to non-TOF (measured as variance reduction) as a function of activity concentration in a 35-cm diameter uniform cylindrical phantom. This measurement was performed on the Super PETT 1 scanner [8]. This figure was originally published in [173] (©1982 IEEE).

Since the first generation of TOF-PET scanners in the 1980s were also being used for high-count rate brain and cardiac studies, it was recognized and shown that this gain in effective sensitivity also increases as the randoms fraction increases [173]. Fig. 10 shows the variance reduction (or the gain in SNR^2^) due to TOF in a 35-cm diameter uniform cylinder as a function of activity concentration [173]. At the lower activity concentration, the gain agrees with the Tomitani [6] estimate, and it increases as the activity concentration (and randoms fraction) increases. In the Appendix, we give a derivation for the variance reduction achieved with TOF-PET, where the reduction depends not only on the TOF resolution but also on the amount of surrounding activity or random coincidences.

The advent of fully 3D PET led to the formulation of the noise-equivalent counts (NEC) metric [174] that includes the effect of scatter and random coincidences on image SNR. For non-TOF PET, it was shown that the image SNR (again for analytic reconstruction) at the center of a uniform cylindrical activity distribution is proportional to the square root of the NEC. With the reintroduction of TOF-PET scanners in the mid-2000s (second generation TOF-PET), the definition of NEC was expanded to include the impact of TOF information [175].

A metric, such as NEC_TOF_ is a useful physical measure to represent global image quality, and in its use of uniform cylindrical phantoms and assumption of analytic image reconstruction it represents a good first measure in estimating the impact of TOF on PET image quality. However, clinical imaging involves patients with heterogeneous activity distribution as well as nonuniform attenuation. More importantly, modern PET scanners, including the second-generation TOF-PET system introduced in the mid-2000s, all utilize iterative reconstruction algorithms that have varying convergence properties that affect resultant PET image quality [130]. In these situations, it is crucial to carefully match the image resolution (iteration number) in order to quantify any reductions in image variance due to TOF resolution.

**Fig. 11.**
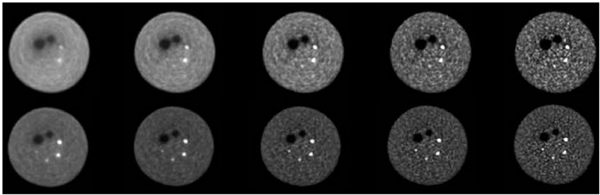
Transverse images from measurements performed with a 35-cm diameter lesion phantom. There are two cold spheres (28, 37 mm) and four hot spheres (10, 13, 17, 22 mm) with 6:1 contrast. Images are shown for iteration numbers 1, 2, 5, 10, and 20, moving from left to right. Data were acquired on the Philips Gemini TF (TOF resolution of 585 ps FWHM). The top and bottom rows show non-TOF and TOF images, respectively. List-mode OSEM with 33 subsets was used for image reconstruction. This figure was originally published in [178] (©2008 SNMMI).

Phantom studies as well as patient data sets have shown that TOF information leads to a faster convergence of lesion contrast [18], [176]–[182]. Fig. 11 shows measured data with hot and cold spheres in a 35-cm diameter phantom as a function of the number of iterations. The data are from a 5-min scan and the images were reconstructed with a list-mode ML-EM algorithm. As the number of iterations increases, lesion contrast improves together with increased noise. However, the convergence rate of contrast is faster with TOF, especially for the 10-mm diameter sphere, indicating that higher contrast is achieved with TOF at an earlier iteration that corresponds to a lower image noise. Faster contrast convergence together with different noise correlations impact lesion detectability performance as described below.

### A. Impact on Clinical Tasks

While the sensitivity or NEC gain metrics provide a good measure of relative gains due to TOF, assigning a single gain factor for TOF does not fully capture the impact on clinical performance and more task-specific metrics are needed to better define the improved performance. Two clinically relevant tasks in oncologic PET are lesion detectability and lesion uptake measurement, both of which have been evaluated over many years in the context of improved performance due to TOF information.

**Fig. 12.**
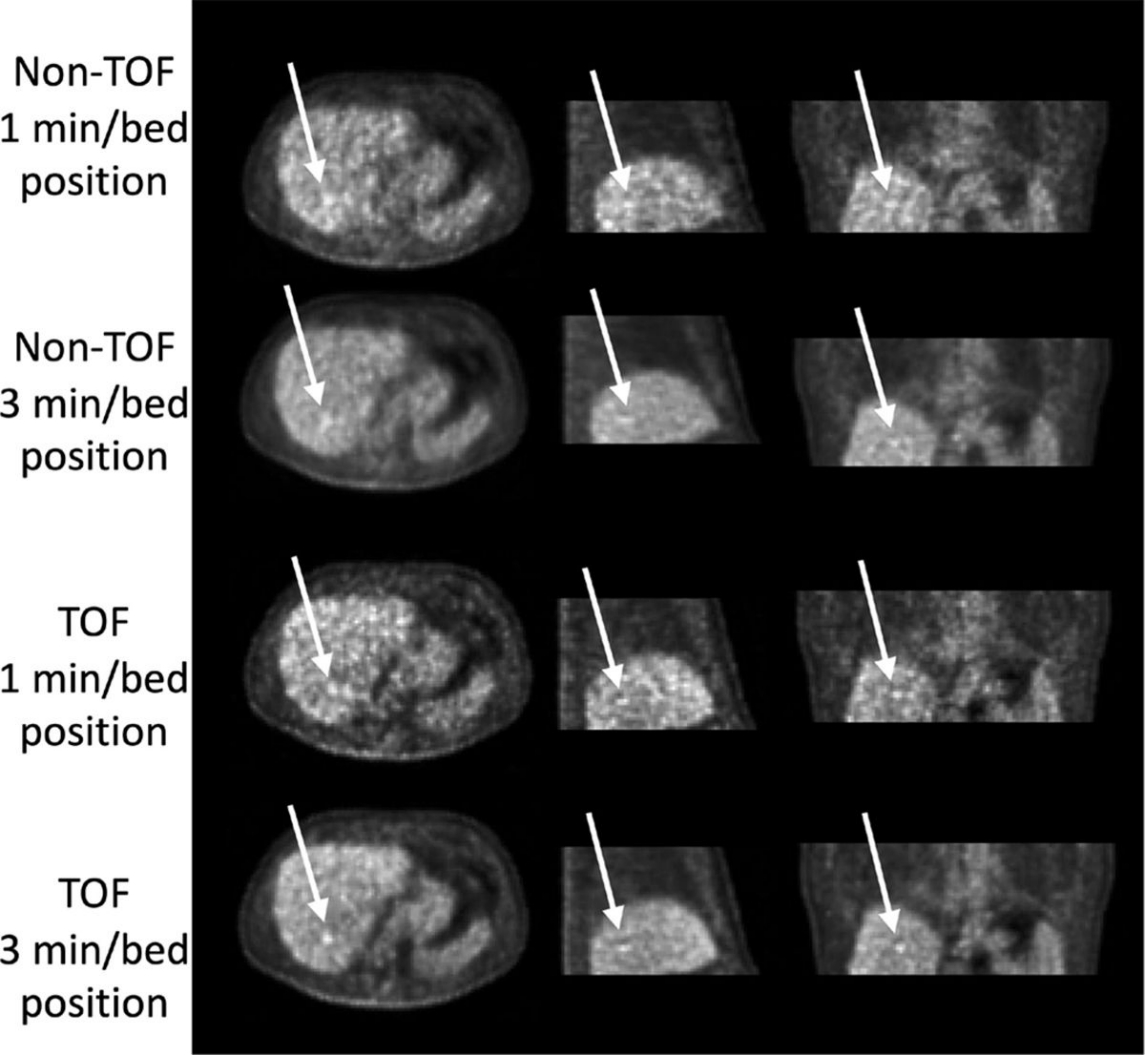
Reconstructed images for a patient study as a function of scan time and TOF or non-TOF reconstruction. Arrows indicate the location of a 1-cm diameter spherical lesion that was synthetically added to the patient data prior to reconstruction. Data were acquired on the Philips Gemini TF (TOF resolution of 585 ps FWHM) and reconstructed using a list-mode OSEM algorithm (using three iterations and 33 subsets in all cases). This figure was originally published in [183] (©2011 SNMMI).

Starting with simulations and measurements of small-lesion detectability in uniform phantoms and realistic clinical patient studies, there exists a significant body of research work evaluating lesion detectability using clinically relevant metrics, such as area under a receiver operating characteristic (ROC) or localized ROC (LROC) curve. Fig. 12 shows representative images from one of these studies, which used data from 100 clinical patients and relied on human observers to perform an LROC evaluation [183]. Lesion data were synthetically added to liver and lung prior to image reconstruction and subsequent reading by human observers. For this image (patient BMI = 28.4) a 3 min/bed position TOF scan shows an improvement in lesion detection and localization.

The overall conclusions from all lesion detectability and lesion uptake measurement studies using clinically relevant metrics are that: 1) for a fixed scan time TOF imaging leads to improved lesion detectability [177], [179], [183]–[185]; 2) imaging times can be shortened with TOF-PET without degrading lesion detectability [183]; 3) gains in lesion detectability increase as the patient or object size increases [177], [179], [183]; 4) lesion detectability performance is more uniform over all patient sizes [183]; and 5) TOF imaging reduces variability in lesion uptake measurement statistically as well as over different organs and different patients [186].

**Fig. 13.**
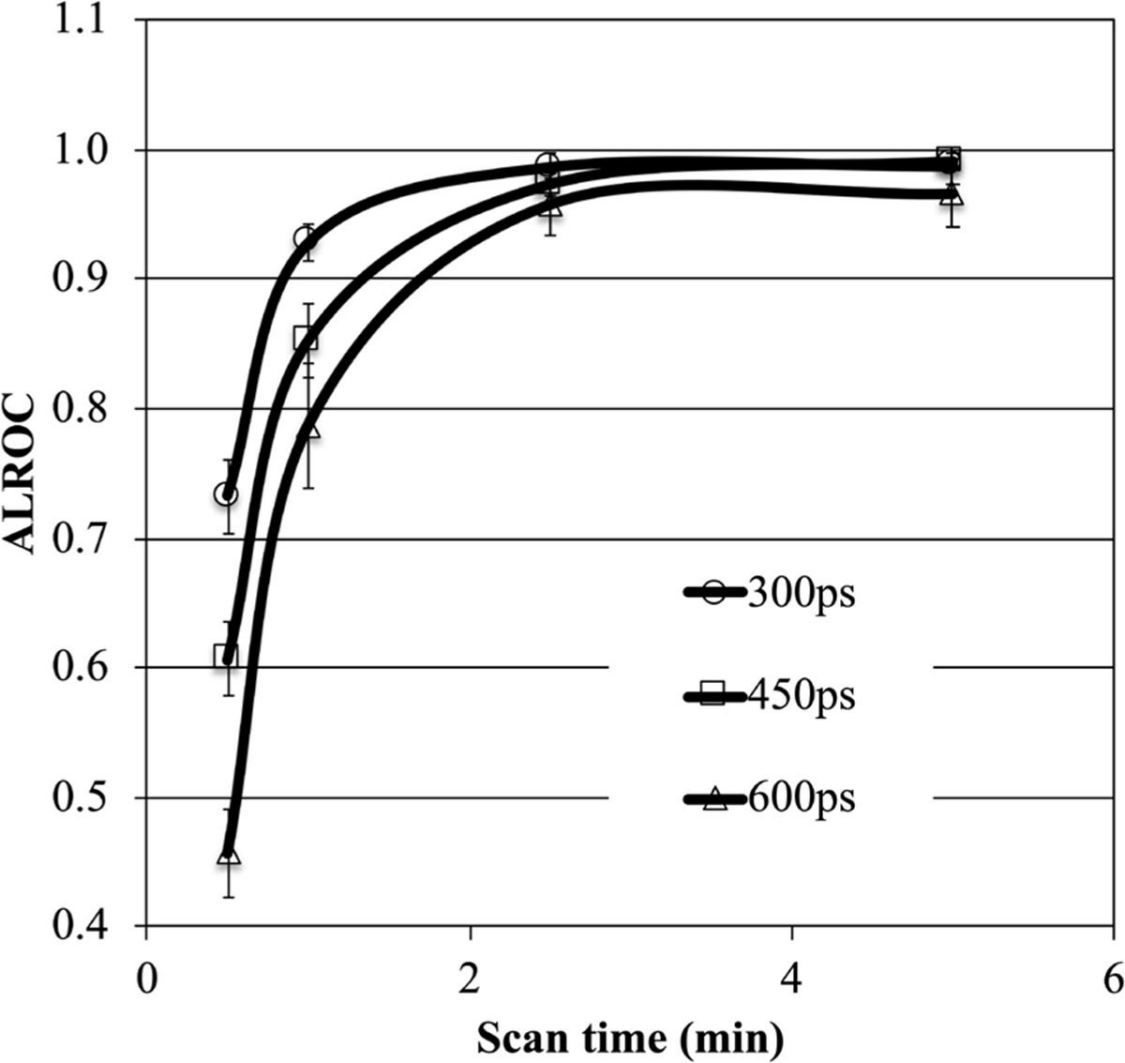
ALROC results for 1-cm diameter spheres in a 35-cm diameter cylindrical phantom with 3:1 relative uptake. Results are shown as a function of scan time for scanners with TOF resolutions of 300 ps, 450 ps, and 600 ps FWHM. All other scanner and imaging characteristics were identical. List-mode OSEM reconstruction with 25 subsets was used and results are shown for the iteration number that produces the maximal ALROC value. Figure derived from data originally presented in [190].

Fig. 13 shows simulation results for the area under the LROC (ALROC) curve values calculated as a function of scan time for 1-cm diameter spheres placed in uniform cylindrical phantom (3:1 uptake ratio). The three curves represent an identical scanner design except for the system TOF resolution. As scan time increases, ALROC reaches a maximum value of 1 for all three scanners. Hence, for long scan times there is no noticeable gain due to TOF since statistical noise is very low, but for shorter scan times the differences are noticeable. Due to the nonlinear nature of the ALROC metric, the gain in ALROC value due to TOF information at a fixed scan time varies, making it hard to assign a fixed gain factor. Alternatively, by comparing data points with similar ALROC values (and < 1), one can estimate the increased scan time necessary in a scanner with a worse TOF resolution. This correlates reasonably well with the expected TOF gain for the NEC or sensitivity metrics.

### B. TOF Versus Other Basic Performance Parameters

Depending on the detector design there can be a trade-off between various performance characteristics of a PET scanner. Two of the more relevant trade-offs for whole-body PET scanner designs are TOF resolution versus spatial resolution, and TOF resolution versus sensitivity. Simulation studies have shown that improved spatial resolution leads to gains in lesion detectability that are similar to those achieved with improved TOF resolution [187], [188]. Improved spatial resolution also reduces partial volume effects and leads to a higher lesion contrast recovery coefficient (CRC) or uptake measurement, but at the cost of increased statistical variability in the measurement. Results from measurements performed on two generations of TOF-PET scanners from the same commercial manufacturer are consistent with these conclusions [189].

Simulation studies have also been performed where lesion detectability is estimated as a function of TOF resolution for identical detector design except for varying crystal thickness [190]. Results in this study showed that a detector with 15-mm thick LSO crystals and 300–450 FWHM ps TOF resolution gives as good a performance as a detector using 20-mm thick LSO crystals with 450–600 FWHM ps timing resolution. Coincidence sensitivity of 20-mm thick LSO is about 33% higher than 15-mm thick LSO, which is similar to the gain in effective sensitivity due to improved TOF resolution (300–450 ps FWHM versus 450–600 ps FWHM). Hence, the crystal volume can be reduced while maintaining similar detectability performance if TOF resolution is improved.

For long axial-FOV PET systems [191], [192], for which cost may be an important consideration in widespread adoption, these results indicate that, with improved TOF resolution, shorter crystals may provide a cost-effective system design. Some of the latest commercial PET/CT system have already demonstrated TOF resolutions in the range of 214–380 ps FWHM [22]–[26] and the PennPET Explorer achieves a TOF resolution of 256 ps FWHM [192], indicating that cost-effective system designs with improved TOF resolution may be feasible.

## V. Future of TOF in Pet

### A. Outlook on TOF-PET Scintillation Detectors

LSO:Ce, LYSO:Ce, and other scintillators in the group of lutetium-based oxyorthosilicates combine excellent timing properties with high density and effective atomic number, making them very suitable for optimizing the effective sensitivity defined in (4). Presently, there appears to be no obvious candidate material with potential to outperform these scintillators in terms of both detection efficiency and time resolution. LaBr_3_:Ce and CeBr_3_, for example, have better values of √*Y*/*τ*_decay_, but significantly lower density. The search for better TOF-PET scintillators is nevertheless ongoing and it cannot be excluded that new materials will be discovered in the future.

Another noteworthy area of research is the development of less expensive TOF-PET scintillators. Ce-doped multicomponent garnets [46], for example, can potentially be produced cost-effectively in the form of scintillating optical ceramics [193]. Another example is the hybrid Cherenkov/scintillation approach, in which the faint but prompt Cherenkov emission in e.g., BGO is utilized to enhance the time-of-interaction estimate, while the much brighter but relatively slow scintillation signal is used for position and energy determination [194]–[196]. An interesting variation on this approach is the combined measurement of Cherenkov photons and charge carriers in wide-bandgap semiconductors [197], [198].

The replacement of PMTs by SiPMs in commercial TOF-PET systems has resulted in a considerable improvement of time resolution in the last ~5 years. SiPMs offer better PDE as well as SPTR values and enable more favorable detector geometries. SiPM developers continue to improve the PDE and SPTR of their devices, which will help to further improve the TOF resolution of PET systems. Yet, some SiPMs already have a PDE of about 60%, so the room for continued improvement is getting smaller.

TOF-PET system manufacturers have exploited the compact form factor of SiPMs to significantly lower the degree of light sharing in their detectors. This reduces the influence of the OTTS on the time resolution (Section II-D). Still, there remains significant potential to further mitigate the three causes of time resolution loss related to optical transfer (Section II-A), even with existing scintillators and SiPMs. This warrants research on new detector geometries that minimize optical transfer time dispersion and/or enable DOI-correction of timestamps (which can be referred to as *time resolution recovery*). Some examples applicable to long crystals are dual-sided readout [199], side readout [200], phoswich approaches [201], [202], and various forms of intercrystal light sharing [203]–[210].

A different approach is the monolithic scintillator detector, which consists of a large (typically several cm^3^) single crystal read out by SiPM array(s) coupled to one or more of its surfaces [211]. The 3D position of interaction in the crystal is decoded from the measured light intensity map(s), while multiple timestamps (typically one per SiPM pixel) are available to estimate the time of interaction. The large amount of spatiotemporal information obtained per event makes it possible to reduce all three causes of OTTS-related time resolution loss discussed in Section II-A, as well as the influence of the photosensor SPTR [28]. The maximum-likelihood interaction-time estimation (MLITE) algorithm by Van Dam *et al.*, for example, can thus be seen as an advanced form of time resolution recovery [212]. It has been demonstrated that monolithic scintillator detectors based on commercially available LYSO:Ce crystals and SiPMs enable sub-200 ps FWHM TOF resolution, excellent spatial resolution, high energy resolution, and correction of parallax errors in clinical PET rings [213], [214].

Through optimization of scintillators, further development of SiPM technology, and research on time resolution recovery methods, a TOF resolution of about 100 ps FWHM, in combination with high detection efficiency, spatial resolution, and energy resolution, appears to be an ambitious but realistic objective for the next-generation of clinical PET systems. Recent simulations indicate that TOF resolutions of 100 ps FWHM or better could also be beneficial in improving the CNR performance of small-animal PET scanners [215].

### B. Outlook on TOF Reconstruction

Further improvement of TOF resolution poses many opportunities but also challenges for image reconstruction in next-generation PET systems. As argued above, it is reasonable to assume that the uncertainty in the TOF direction will remain much larger than the uncertainty caused by photon acollinearity and finite detector size in the next generation(s) of clinical PET systems. This implies that the idea of “reconstructionless” PET (in which a simple TOF back-projection of precorrected data could be considered as reconstruction) will not yet be feasible in the foreseeable future. Instead, iterative reconstruction techniques using a detailed forward model capturing the physics of TOF coincidence detection will remain the method of choice.

Further improvement of the TOF resolution will naturally increase the information content of each measured coincidence event. This additional information will further improve the achievable bias versus noise trade-off, the accuracy and stability of joint estimation algorithms for activity and attenuation, and the image quality achievable from systems with limited angular coverage as discussed in Section III.

It is important to keep in mind that the quality of any model-based iterative reconstruction is only as good as the quality of the applied forward model. For systems with improved TOF resolution, this means that the modeled accuracy of all factors influencing the TOF measurement must be improved in a similar way. These factors include precise and stable calibration of crystal coincidence timing offsets, determination of the (LOR-dependent) TOF resolution, precise modeling of the (LOR-dependent) TOF kernel, and an accurate TOF scatter estimation. For current PET systems, the uncertainty on the TOF measurement could be well modeled as Gaussian, and the same uncertainty could be assumed for all events. In some new detector designs, this is no longer the case [216]. For example, detectors using the hybrid Cherenkov/scintillation approach may have a non-Gaussian TOF-kernel [217]. The same may be the case for detectors based on metamaterials [43], [218], where different events can have a very different timing resolution. For such PET systems, the reconstruction model will have to account either for the average non-Gaussian uncertainty or for the timing uncertainty associated with each event, if such information is available [216], [217].

Improvement of system TOF resolution is not the only factor that is expected to enable better image quality in future PET systems. Advances in the PET detector technology might allow the use of additional properties of the detected coincidences, e.g., improvements in the detector energy resolution might enable using the energy information of the two detected photons to better model or directly reject scattered coincidences. Moreover, techniques that allow to deduce information about the incidence direction of the incoming photons, e.g., via an analysis of intracrystal (layer) Compton scattering, might be feasible in future [219]. To achieve an optimal and stable quality of future PET reconstructions, the joint benefit of all those factors should be considered instead of solely focusing on the improvement of TOF resolution.

### C. Improving Time Resolution: The Quest for 10 ps PET

**Fig. 14.**
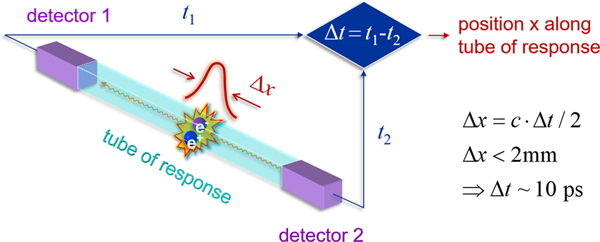
Time resolution in the order of ~10 ps FWHM would enable direct event localization in PET. This figure was originally presented in [38] and [39].

In principle, direct localization of the point of annihilation photon emission would become possible if detectors with a time resolution in the order of ~10 ps could be developed (Fig. 14). About a decade ago, Schaart *et al.* [38], [39] argued that this would be very difficult to achieve with lanthanide-doped scintillators and that a new method of annihilation photon detection would be needed to reach this goal.

Recently, a TOF resolution of ~30 ps FWHM has been achieved with a pair of MCP-PMTs in which the photocathode was deposited on a 3.2-mm thick lead glass entrance window acting as a Cherenkov radiator [220]. All events except those with the highest Cherenkov photon count were rejected. While the authors acknowledge that the correspondingly low detection efficiency does not satisfy the requirements of a clinical PET detector [see (4)], their experiment shows that the physics of positron annihilation allow a TOF resolution in the order of tens of ps.

A variety of researchers, among others from the high-energy physics community, are currently advocating the so-called “10 ps challenge.” A plethora of novel approaches are under investigation, based on, for example, prompt emissions, such as Cherenkov and hot-intraband luminescence, or enhanced luminesce resulting from quantum-confinement in nano- and metamaterials [43], [218]. Also under investigation are systems in which lasers are used to actively probe transient phenomena caused by the absorption of annihilation photons [221], [222]. While some of this research is very exciting from the physics perspective, pushing the limits of TOF resolution only becomes meaningful when it contributes to the improvement of PET imaging performance [223]. Thus, it is hoped that a new technology will emerge from this research that not only enables ultraprecise TOF determination but also fulfills all other requirements of a good PET detector.

Moreover, some people might think of a 10 ps PET scanner as a system that allows for “reconstruction-less” PET imaging. We would like to emphasize that quantitative PET imaging will certainly require more than a simple TOF back-projection in such systems. For example, random and scattered coincidences still have to be modeled and corrected for. The latter is traditionally done in an iterative way and requires knowledge of the distribution of activity and attenuation in image space. It has also been hypothesized that ~10 ps TOF information could be used in iterative reconstruction to improve the spatial resolution of high-resolution systems beyond the conventional limit imposed by the crystal pitch [224], though other effects, such as detector scatter may still limit such gains. Finally, due to the limited number of acquired events, some sort of noise suppression has to be included in the image generation process (either during or post reconstruction).

Taking into account the current rapid evolutions in the field of inverse problems and machine learning that will very likely also impact the way PET images are generated from PET raw data, it is nearly impossible to predict how exactly and where all those corrections will be implemented once systems with TOF resolutions comparable to the crystal size become available. However, as long as coincident events include random and scatter coincidences, some kind of image reconstruction beyond simple TOF back-projections will be required to obtain quantitative images with reasonable bias-noise trade-offs. Such future reconstruction algorithms might be very different from the model-based iterative algorithms (e.g., TOF-OSEM) that are used in current PET systems.

In summary, for ~10 ps TOF-PET to emerge as a clinical imaging modality one day, major innovations in both detector technology and image reconstruction methods will be required. If successful, these combined developments could open up unprecedented possibilities for improving the sensitivity, resolution, and quantitative imaging performance of PET scanners.

### D. TOF Versus Total-Body Coverage

In the last two years we have seen the introduction of total-body PET (TB-PET) scanners [191], [192]. These systems are long AFOV systems (up to 194 cm long) that provide both a large gain in sensitivity due to a high geometric efficiency and an ability to simultaneously measure dynamic uptake of radiotracers over a large axial coverage of the body [225]. While providing exquisite images with potential for transforming PET research and patient care [226], [227], an obvious dilemma is imposed by the increased cost of such systems. In particular, the use of Lu-based scintillators is expected to be a main factor driving the costs of these systems. However, given the extremely high geometric sensitivity of these systems, a natural question to ask is whether there is any need for TOF capability.

Since the gain in imaging performance due to TOF acts like a gain in effective sensitivity (4), one could potentially use an inexpensive scintillator, such as BGO that has a higher detection efficiency than Lu-based scintillators and achieve most of the benefits (long AFOV and high sensitivity) of TB-PET imaging [228]. However, some of the other advantages of TOF-PET, such as joint estimation of emission and attenuation and robustness of data, will be lost, unless researchers succeed in utilizing the Cherenkov emission that occurs in BGO for transforming this material into a TOF-capable detector.

Another approach could be to utilize the advantages of TOF for reducing the amount of Lu-based scintillator used in a TB-PET system. For instance, one could reduce the crystal thickness (and hence total crystal volume) while potentially improving TOF resolution and achieving sufficient effective sensitivity for TB-PET imaging applications [190], [229], [230]. Alternatively, the robustness of PET data with improved TOF resolution allows the possibility of using Lu-based detectors in a sparse arrangement (gaps, axially and/or trans-axially) [231]–[236], allowing reduction in detector cost while achieving longer axial FOV coverage. In fact, the prototype configuration of the PennPET Explorer [192], [226] has gaps in each ring due to current electronics limitations, leading to a data loss of 30 percent in each ring. Despite this loss of data, the studies demonstrate high quality, artifact-free images can be generated. Both these ideas of leveraging TOF benefits to reduce crystal volume will lead to a less expensive TB-PET design that will have lower sensitivity but provide a long axial FOV for multiorgan dynamic imaging.

Hence, despite the large gains in intrinsic system sensitivity achieved with longer AFOV systems, TOF capability will lead to additional performance gains and/or enable development of relatively inexpensive long AFOV systems. Furthermore, the ability to perform joint emission and attenuation estimation with TOF information provides a unique capability to perform high quality, ultralow-dose PET-only studies in patients, which is important in situations, such as pediatric imaging or serial imaging of a patient.

## VI. Conclusion

In the last ~15 years, the parallel development of TOF-capable PET instrumentation and image reconstruction methods that exploit the additional information per count have greatly advanced the state of the art in clinical molecular imaging. This has resulted in tangible benefits for physicians and patients. In addition to further improvements in time resolution, we anticipate that the new possibilities offered by TOF, such as combined estimation of emission and attenuation, or artefact-free reconstruction of limited-angle PET images, will spur the development of multimodality and organ-specific systems, among others.

It will be interesting to see how far the field will be able to continue pushing the limits in timing performance and how this will eventually affect clinical PET imaging, especially in comparison to other recent developments, such as TB-PET imaging. Even though it is hard to predict what the field of molecular imaging will look like in another ~15 years, we believe that the role of PET in (personalized) clinical medicine will continue to grow. It is, therefore, a very interesting time for young researchers to join the field and make their own, perhaps unexpected, contributions to the further advancement of this important imaging modality.

## Figures and Tables

**TABLE I:** Overview of TOF-PET Scintillators and Their Properties.

Scintillator	*ρ* (g cm^−3^)	*Z*_eff_	*Y* (keV^−1^)	*τ*_decay_ (ns)	Energy resolution (% FWHM)
BaF_2_	4.9	54	1.3–1.4	0.8	8
CeBr_3_	5.2	46	57–66	17	4
CsF	4.6	52	1.9–2.0	3	~20
LaBr_3_:Ce	5.1	45	64–76	16	3
LFS-3	7.3	65	~38	35–40	8
LGSO-Fast	7.2	66	~34	30–34	8
L(Y)SO:Ce	7.1–7.4	65–66	26–34	38–44	8
L(Y)SO:Ce,Ca	7.1–7.4	65–66	32–40	31–39	8

Data were taken from the publications cited in Section II-B. Uncertainties are in the order of one last digit, unless (a) the value is preceded by a tilde, in which case the uncertainty is larger, or (b) a range of values is given, in which case this range reflects the spread encountered in the papers cited. The energy resolution is given for 662-keV photon irradiation.

**Fig. 15.**
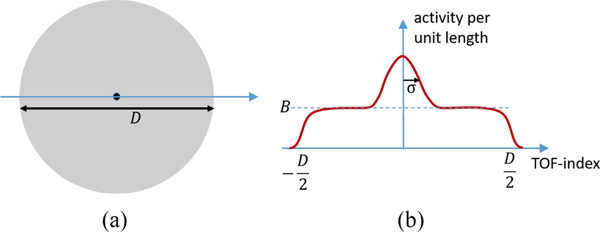
(a) Disk phantom with a hot spot in the center. (b) TOF profile, as measured along an LOR through the hot spot, is a blurred version of the true profile.

**Fig. 16.**
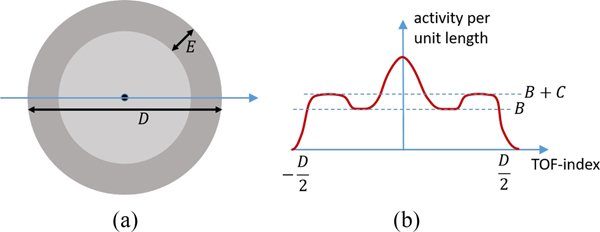
Modified disk phantom with a ring of increased activity of width *E* and excess activity per unit length *C*.

## Appendix

This Appendix presents a derivation of the gain in variance produced by TOF, by considering only a single LOR. Consider a disk with diameter *D*, filled with a uniform activity (see Fig. 15). In the center is an infinitely small spot with slightly increased activity. Attenuation is ignored. Because of symmetry, all LORs through the center have the same expected measurement. Therefore, we compute the signal and variance, provided by a single LOR, for detecting presence of the spot. *B* denotes the activity per unit length along the LOR, *S* is the total excess activity of the spot at position *x* = 0. The total activity along the LOR equals *BD*. We assume *S* << *BD*.

A non-TOF measurement with a detector pair produces a single value for the LOR. If the spot is present, the expectation of the measurement equals *BD*+*S*. If the spot is absent, it equals *BD*. Therefore, the signal obtained from the measurement equals *S*. The variance of the measurement equals *BD*, because the data are subject to Poisson noise and *S* << *BD*.

In the TOF-case, the detector pair produces a profile along the LOR, which is a blurred and noisy version of the true profile. The expectation of the profile with spot present equals

(10) $$P(x)=B+\frac{S}{\sqrt{2\pi \sigma }}e^{-\frac{x^{2}}{2\sigma^{2}}}$$

where *x* is the 1D coordinate along the LOR and *σ* is the TOF uncertainty. This expression only holds for the central part of the profile, but since *D* >> σ, we will only need that central part. Assuming that Poisson noise can be well approximated as Gaussian noise, the optimal way to test if the hot spot is present in the profile, is to apply a prewhitening matched filter. With the usual assumption that the noise is uncorrelated, no prewhitening is needed and the matched filter equals the expectation of the difference between signal present and signal absent measurements, times an arbitrary constant. Setting the filter to $F(x)=\sqrt{2}\exp\left(-x^{2}/2\sigma^{2}\right)$ and applying it to the difference between the spot present and the spot absent measurements produces the signal

(11) $${\mathrm{signal}}_{\text{TOF}}=\int_{-\infty }^{\infty }(P(x)-B)F(x)dx\;\;=\frac{S}{\sqrt{\pi }\sigma }\int_{-\infty }^{\infty }e^{-\frac{x^{2}}{\sigma^{2}}}dx=S.$$

The associated variance is given by

(12) $${\mathrm{var}}_{\text{TOF}}=\int_{-\infty }^{\infty }B{\left(F(x)\right)}^{2}dx=2B\sqrt{\pi }\sigma .$$

For both cases, the signal equals *S*. The gain in variance equals

(13) $$\frac{{\text{var}}_{\text{nonTOF}}}{{\text{var}}_{\text{TOF}}}=\frac{BD}{2B\sqrt{\pi }\sigma }=\sqrt{\frac{2\ln2}{\pi }}\frac{D}{\text{FWHM}}=0.66\frac{\text{D}}{\text{FWHM}}.$$

where we used $\text{FWHM}=\sqrt{8\ln2}\sigma$. This result is identical to that obtained by Tomitani for the variance gain in the center of a uniform cylinder, reconstructed with FBP [6].

This same analysis can easily be applied to a modified disk, in which an excess activity per unit length *C* is added to the outer ring with width *E* of the disk (see Fig. 16). For the TOF-case, nothing changes, because the center of the profile along the LOR is not affected. But for the non-TOF case, the total activity along the profile, and therefore the variance of the measurement, increases to *BD* + 2*EC*. Thus, the gain in variance due to TOF now becomes

(14) $$\frac{{\text{var}}_{\text{nonTOF}\;}}{{\text{var}}_{\text{TOF}\;}}=0.66\frac{D+2EC/B}{\;\text{FWHM}\;}.$$

Thus, if *C* is positive, i.e., if the spot is surrounded by more activity, then the gain obtained from TOF is even higher than the Tomitani prediction. If *C* is negative, the gain is lower.

In a similar way, the effect of randoms can be incorporated in (14). The randoms contribution is uniform over the TOF bins. Let *D*_FOV_ be the diameter of the FOV, *R* the number of randoms per unit length and, therefore, *RD*_FOV_ the total non-TOF randoms contribution, and *β* = *D*/*D*_FOV_. Then, (14) becomes

(15) $$\frac{{\text{var}}_{\text{nonTOF}}}{{\mathrm{var}}_{\text{TOF}}}=\frac{BD+RD_{\text{FOV}}}{2\sqrt{\pi }(B+R)\sigma }=0.66\frac{D}{\text{FWHM}}\frac{B+R/\beta }{B+R}$$

which predicts that TOF reduces the variance more when the PET FOV is larger, since the non-TOF acquisition will collect more randoms.
